# HTCC-Modified Nanoclay for Tissue Engineering Applications: A Synergistic Cell Growth and Antibacterial Efficiency

**DOI:** 10.1155/2013/749240

**Published:** 2013-08-12

**Authors:** Majid Aliabadi, Roya Dastjerdi, Kourosh Kabiri

**Affiliations:** ^1^Department of Chemical Engineering, Islamic Azad University, Birjand Branch, P.O. Box 97178-131, Birjand, Iran; ^2^Textile Engineering Department, Yazd University, P.O. Box 89195-741, Yazd, Iran; ^3^Iran Polymer and Petrochemical Institute (IPPI), P.O. Box 14965-115, Tehran, Iran

## Abstract

This paper deals with the synthesis of a biocompatible chitosan ammonium salt N-(2-hydroxy) propyl-3-trimethylammonium chitosan chloride (HTCC) and using it in montmorillonite ion-exchange process. HTCC-modified montmorillonite (Mt) with different chemical ratios was successfully synthesized, and their characteristics have been verified by XRD and FTIR analyses. Produced samples have been evaluated in terms of antibacterial efficiency and biocompatibility (cell culture test). Antibacterial efficiency of synthesized HTCC/Mt samples has been confirmed against both gram negative bacteria (*Escherichia coli*) and gram positive bacteria (*Staphylococcus aureus*). The results disclosed that the antibacterial efficiency of HTCC-modified montmorillonite was unexpectedly even more than HTCC. This excellent synergistic effect has been referred to entrapping bacteria between the intercalated structures of HTCC-modified montmorillonite. Then HTCC on clay layers can seriously attack and damage the entrapped bacteria. An extraordinary biocompatibility, cell attachment, and cell growth even more than tissue culture polystyrene (TCPS) have been recorded in the case of this novel kind of modified clay. Due to existing concerns about serious and chronic infections after implant placement, this natural-based bioactive and antibacterial modified clay can be used in electrospun nanofibers and other polymeric implants with promising mechanical properties for tissue engineering applications.

## 1. Introduction

Recently, using nanostructures in biomedical application has been increasingly interesting [1–6]. One of the weak spots, in the case of using polymeric implants in tissue engineering application, especially in bone cement scaffolds, is their insufficient mechanical strength [7]. Using clay nanocomposites is a good alternative especially regarding their brilliant strength, modulus, and dimensional stabilities [8–12]. However, the differences between surface energies of mineral clay and polymeric surfaces cause a major difficulty in the clay intercalation as well as nanocomposite processing. Then oregano surface modification of clay is necessary for preparing the polymeric clay nanocomposites [13]. However, the most common ion exchange reactions based on using low molar mass intercalants like alkyl ammonium salts, for example, hexadecyltrimethylammonium bromide [14, 15] and alkyl amines, for example, octadecylamine [16, 17] are not suitable for biological applications due to their toxicity [15, 18]. Consequently, developing a nontoxic, biocompatible, and efficient nanoclay modification for tissue engineering applications is a topic of high interest. On the other hand, control of some serious and chronic infections after implant placement is another challenge in tissue engineering [19]. Therefore, this research aimed to synthesis an innovative chitosan-derivative-modified montmorillonite to develop natural-based biocompatible and antibacterial nanoclay for tissue engineering applications. Chitosan is a well-known biopolymer used for biomedical applications [20–22]. However, antibacterial activity of chitosan is limited to acidic conditions due to the protonation of the amino groups [23]. Therefore chitosan cannot provide the sufficient antibacterial activity in physiological conditions (pH 7.4) [24]. 

However, HTCC (N-(2-hydroxy) propyl-3-trimethylammonium chitosan chloride) is already well known for its better antibacterial properties overall a wide range of pH [23]. However, neither investigation on the possibility and efficiency of using HTCC for ion-exchange reaction with montmorillonite nor biological or antibacterial activity of the HTCC-modified montmorillonite has been reported in the literature so far.

## 2. Materials and Methods

### 2.1. Materials

Natural montmorillonite, Cloisite Na^+^ was supplied by Southern Clay Company; glycidyltrimethylammonium chloride (GTMAC) was purchased from Fluka, chemical company. Chitosan with deacetylation degree of 82% was purchased from Chitotech Co., Iran. Acetone, methanol, ethanol, and acetic acid were purchased from Merck Chemical Company and used without more purification.

### 2.2. Methods

#### 2.2.1. HTCC Synthesizing

21.3 mL glycidyltrimethylammonium chloride (GTMAC) was added to a reactor containing 6.0 g chitosan in 60 mL distilled water stirred at 85°C, in three times with 2 h intervals (7.1 g GTMAC per 2 h). After the third time adding GTMAC, the reaction condition was fixed 4 h more to complete the reaction (then the total reaction time was 10 h). Then, the obtained transparent yellowish solution was filtered and precipitated in cold acetone. The white and gel like precipitated product was aged (kept) in cold condition for 24 h. Then the solid material was dissolved in methanol and subsequently precipitated in mixture of acetone/ethanol solution with a ratio of 4/1. The white solid material was separated and then extracted with acetone by Soxhlet for 48 h. The material was dried in 50°C for 24 h. The value of 0.984 was obtained for the degree of quaternization (DQ) of HTCC. DQ was evaluated by conductometeric titration at 20°C following Mivehi et al. [25]. After confirming the successful HTCC synthesizing by FTIR, the powder was used for montmorillonite modification process in the ion exchange reaction. 

#### 2.2.2. Ion Exchange Reaction

The process was performed following the previous paper [26] summarized as follows. One gram of montmorillonite was dispersed in 50 mL distilled water. Different ratios of HTCC 0.5, 1, and 2 g were separately dissolved in 312 mL of 1% v/v acetic acid aqueous solution. The montmorillonite dispersion and each HTCC solution were then poured in an Erlenmeyer flask. The mixture was heated for 9 h at 70°C. After the heat treatment, the mixture was centrifuged and washed with 1% acetic acid solution and water. Each product was dried in an air-circulating oven at 60°C for 6 h; then it was ground. The produced samples have been introduced in Table 1.

### 2.3. Characterizations

#### 2.3.1. FTIR Spectroscopy

FTIR spectra of dried samples were recorded by an ABB-Bomen MB-100FT-IR spectrophotometer, with the range of 4000–400 cm^−1^ with 2 cm^−1^ resolution.

#### 2.3.2. XRD Analysis

X-ray diffraction of samples was obtained employing X-ray diffractometer (Zimenss D5000) at wavelength Cu-K_*α*_ = 1.54 Å operating at a voltage of 40 kV and a current of 30 mA at the rate of 2°/min in the range of diffraction angle 2–10.

#### 2.3.3. Evaluation of Antibacterial Efficiency

The antibacterial activities of HTCC-modified montmorillonite with different ratios, chitosan, and unmodified montmorillonite powder were evaluated according to a modified version of ASTM E2149 standard. The protocol was briefly as follows. 0.02 g of each sample was dispersed in the 20 mL TPS (tryptic soy broth) in a flask with a cell concentration of 2 × 10^4^ colony forming units per milliliter (CFU/mL). The flask was then shaken at 200 rpm on a rotary shaker at 37°C for 18 h. A serial dilution of each test solution was prepared and spread onto an agar plate. After 48 h. of incubation at 37°C, the number of colonies formed on the agar plate was counted; accordingly, the antibacterial efficiency was calculated based on the average reduction percentage of tested bacteria resulted from triplicate evaluations. The percentage of antibacterial activity was calculated by (1). Consider the following:

(1) $$\begin{array}{c} R\left(\%\right)=\frac{A-B}{A}\times 100, \end{array}$$

where *B* is the number of bacteria recovered from the inoculated test specimen in the flask including each produced sample and *A* is the number of bacteria according to “*B*” conditions without antibacterial samples, considered as the control.

#### 2.3.4. Biological Interactions

Biocompatibility of different produced samples was evaluated using human bone marrow mesenchymal stem cells (BMMSCs). The cells were grown in Dulbecco's modified Eagle medium (DMEM) at 37°C with a 5% CO_2_ atmosphere for several days until cells were confluent. Then cells were harvested and resuspended in DMEM containing 10% fetal bovine serum and 1% penicillin/streptomycin. Total number of cells was determined using a hemocytometer, and 5,000 cells were seeded into each well of 24-well plates. The amount of each samples being assayed (e.g., HTCC, montmorillonite (Mt), different HTTC/Mt samples, etc.) was adjusted to obtain the concentration of 500 ppm in each test. The plates were then incubated for various time points. After the desired time for each well, 15 *μ*L of a solution of 5 mg/mL of 3-(4,5-dimethyl-2-thiazolyl)-2, 5-diphenyltetrazolium bromide (MTT) in PBS was added to the well. The plates were incubated for 3 hours to allow sufficient time for the conversion of the MTT dye (yellow liquid) to the water-insoluble formazan derivative, 1-(4,5-dimethylthiazol-2-yl)-3, 5-diphenylformazan (blue solid) by the mitochondrial dehydrogenases in the living cells. Blue crystals were observed, and the medium was removed from each well by aspiration. The crystals were dissolved by adding 100 *μ*L of dimethylsulfoxide to each well. Viable cell was spectrophotometrically determined using an Eliza reader by measuring the absorbance at 570 nm. The absorbance values were averaged for triplicate tests of each sample at each time.

## 3. Results and Discussions

### 3.1. FTIR Spectroscopy

**Figure 1:**
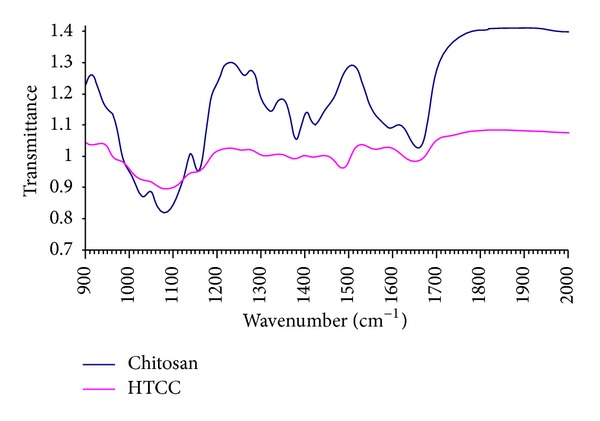
FTIR spectroscopy spectrum of chitosan and HTCC.

Synthesis of chitosan ammonium salt has been confirmed with an initial qualitative test by its solubility in water in the variety range of pH. Only when the amino groups of chitosan are protonated in acidic conditions, it can be dissolved in water. Then chitosan dissolves in water only in acidic conditions, while HTCC solubility is not depending on pH [23]. Therefore solubility in water in the variety range of pH is a sign of successful HTCC synthesis. The synthesizing HTCC has been also verified by studying normalized Fourier transform infrared (FTIR) spectroscopy spectrum evidence (Figure 1). Two peaks on 1640 cm^−1^ and 1480 cm^−1^ associated to C=O stretch of the secondary amide and C–H bending of trimethylammonium, respectively [23], are the HTCC characteristics. Disappearing of the N–H bending peak of the primary amine at 1595 cm^−1^ because of changing the primary amine to the secondary amine and the peak at 1657 cm^−1^ corresponding to C=O stretching of amide groups are the signs of changing the NH_2_ groups of chitosan with 2-hydroxy propyl-3-trimethylammonium chloride groups. This evidence deduced successful HTCC synthesis.

### 3.2. XRD Analysis

**Figure 2:**
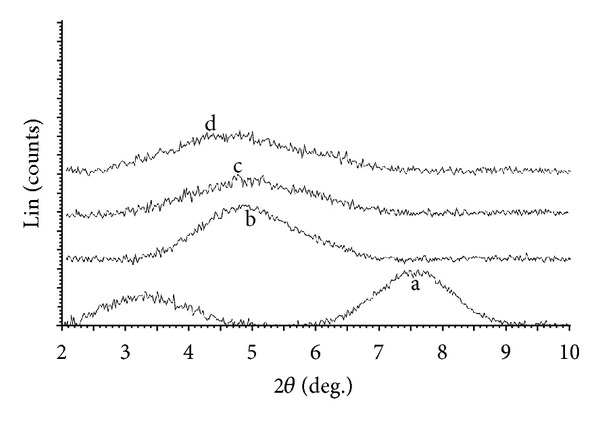
XRD patterns of modified montmorillonite as compared to natural montmorillonite, (a) unmodified montmorillonite, (b) HTCC/Mt = 1/2, (c) HTCC/Mt = 2/2, and (d) HTCC/Mt = 2/1.

XRD patterns of modified montmorillonite samples as compared to natural montmorillonite (unmodified montmorillonite) were demonstrated in Figure 2. XRD analysis is the best method to investigate the efficiency of nanoclay modification [13]. The modification process is necessary for producing intercalated and exfoliated polymeric nanocomposites, in fact, due to the differences between surface energy of inorganic materials and the polymeric matrix [27] intensified by hugely enhanced specific surfaces of the nanolayered silicates, the surface modification is necessary for mixing nano-clay with polymeric matrix [14]. The surface modification occurs by the cation (Na^+^) exchange reaction between the natural montmorillonite, Cloisite Na^+^ used in this research, and intercalant [28]. Alkyl ammonium salts, for example, hexadecyltrimethylammonium bromide [14, 15] and alkyl amines, for example, octadecylamine [29] are the most common family of intercalants. However, these intercalants are mostly toxic and consequently cannot provide a nanocomposite substrate prone to cell growth for biological and tissue engineering applications [15, 18]. However, Figure 2 confirmed the successful cation-intercalant ion exchange reaction using HTCC aimed in this research for the surface modification of nano-clay. The noticeable shift of 2*θ* reflections from 7.6 on the unmodified montmorillonite to 4.7–4.8 for HTCC modified montmorillonite verified achieving a good intercalation. This shift referred to the increased interlayer space from 11.6 Å for unmodified montmorillonite to about 18.8 Å for modified species.

### 3.3. Antibacterial Efficiency

**Figure 3:**
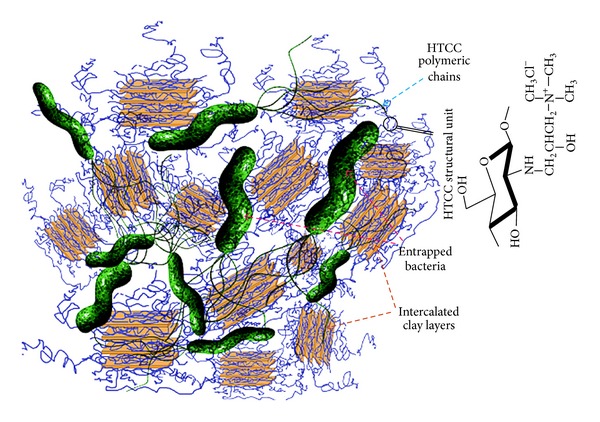
Schematic of the synergistic antibacterial efficiency mechanism, entrapping bacteria between the intercalated HTCC/Mt structures.

**Figure 4:**
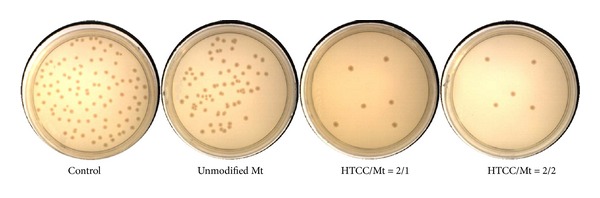
Results of designed qualitative test for comparing the antibacterial efficiency of some samples against *E. coli. *

Among the different nanostructures with antibacterial activity, silver [30–35], TiO_2_ [36–43], and ZnO [44, 45] nanostructures are the most safe and common nanomaterials. Numerous research papers have focused on modification of materials with these nanostructures [46–48]. However, ZnO and TiO_2_ are not proper for implants due to the need of UV irradiation for their activities. Some evidence about antibacterial activity of some montmorillonite species has been also reported [8]. Antibacterial activity evaluation against *Escherichia coli* ATCC 25922 has been reported in Table 2. Although only 32% antibacterial efficiency has been recorded on unmodified montmorillonite sample, montmorillonite modification with HTCC resulted in improving the bioactivity of samples. The optimum value has been obtained on sample produced from equal mass ratio of HTCC and montmorillonite (HTCC/Mt = 2/2). Unexpectedly, the antibacterial efficiency of these samples was even more than pure HTCC. Therefore the antibacterial activity of HTCC-modified montmorillonite was more than both montmorillonite and HTCC. This synergistic effect can be explained as follows. The bacteria are attracted to attach on the intercalated structure of HTCC-modified montmorillonite. They are attracted to anchor into the intercalated structure of HTCC-modified montmorillonite by their flagella. Then, the attached bacteria faced to the HTCC attack on nanoclays layers. This provides an opportunity for HTCC on nanoclay layers to seriously damage the entrapped bacteria before they can release their flagella and escape. Moreover, when bacteria want to escape, they will encounter other intercalated structure of HTCC-modified montmorillonite as the new physical barriers. In this way, they will be more limited and seriously damaged before they can escape. This has been schematically demonstrated in Figure 3. Results of designed qualitative test for comparing the antibacterial efficiency of some samples were also exhibited in Figure 4.

The antibacterial efficiency against *Staphylococcus aureus* ATCC 29213 reported in Table 2 also confirmed the results. An excellent antibacterial efficiency against *Staphylococcus aureus *has been recorded on both HTCC/Mt = 2/2 and HTCC/Mt = 2/1.

### 3.4. Biological Interactions

**Figure 5:**
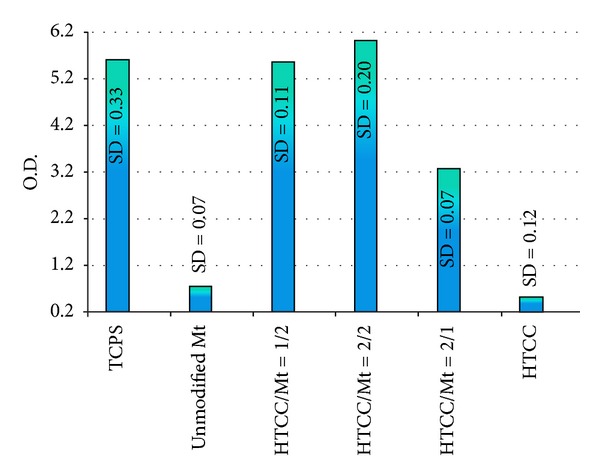
Cell culture test results on 96 h postseeding samples.

**Figure 6:**
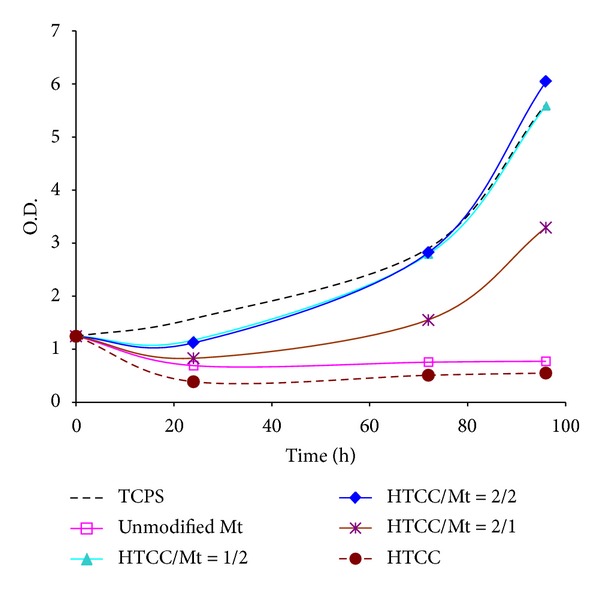
Cell culture test results by time.

Cell culture test results on 96 h postseeding samples were showed in Figure 5. A very good cell growth potentiality has been recorded on the HTCC-modified nano-clay samples. According to the results, a synergistic effect has been observed on all of the modified nano-clay samples. Cell growth on modified clay samples has been improved as compared to both HTCC and montmorillonite. About 6–11 times increases in the cell growth as compared to HTCC and about 4–8 times increases in the cell growth as compared to unmodified montmorillonite have been recorded. Sample HTCC/Mt = 2/2 with about 11 times (about 999%) cell growth improvement as compared to HTCC demonstrated the best cell growth improvement. As it has been well known, tissue culture polystyrene (TCPS) is one of the most prone substrate to cell attachment and cell growth and widely used as a control in cell culture tests [49]. Interestingly, cell growth on the sample HTCC/Mt = 2/2 was even more than the tissue culture polystyrene after 4 days seeding. The cell growth by time has been also compared in Figure 6. The best biocompatibility and antibacterial efficiency against both kinds of bacteria have been recorded in the case of this sample (HTCC/Mt = 2/2).

## 4. Conclusions

An innovative chitosan-derivative-modified montmorillonite (HTCC-modified montmorillonite) with an excellent biocompatibility, cell growth, and antibacterial efficiency for tissue engineering applications was synthesized and evaluated in the terms of biological and microbiological activities. To this end, at first, chitosan ammonium salt N-(2-hydroxy) propyl-3-trimethylammonium chitosan chloride (HTCC) was successfully synthesized and evaluated by FTIR spectroscopy. The montmorillonite ion-exchanged reaction has been conducted with different ratios of HTCC and montmorillonite. Desirable intercalation was confirmed by XRD analyses. A brilliant synergistic effect on antibacterial activity even more than pure THCC was recorded for the produced HTCC-modified montmorillonite samples. This is referred to enhanced antibacterial efficiency of HTCC on entrapped bacteria between the intercalated structures of HTCC-modified montmorillonite. An exceptional cell growth of samples was also confirmed by cell culture experiments.

## Figures and Tables

**Table 1:** Introducing of different samples.

Samples	The material ratios		
	HTCC (g)	Mt (g)	Chitosan (g)
HTCC	Pure	—	—
Montmorillonite	—	Pure	—
HTCC/Mt = 1/2	0.5	1	—
HTCC/Mt = 2/2	1	1	—
HTCC/Mt = 2/1	2	1	
Chitosan/Mt = 2/1	—	1	2
Chitosan	—	—	Pure

**Table 2:** Antibacterial efficiency of different samples.

Samples	*Escherichia coli *		*Staphylococcus aureus *	
	NO.^1^ × 10^−5^	*R*%	NO.^1^ × 10^−5^	*R*%
Control	200	0	151	0
HTCC	68	66	8	94.43
Montmorillonite	136	32	22	85.53
HTCC/Mt = 1/2	114	43	55	63.51
HTCC/Mt = 2/2	11	94.5	4	97.34
HTCC/Mt = 2/1	13	93	1	99.01
Chitosan/Mt = 2/1	108	46	71	52.69
Chitosan	119	40.5	72	52.44

^1^Number of grown bacteria per mL after the incubation time (100000 times diluted).
